# Distinct molecular profiles of indeterminate and malignant thyroid nodules in patients under 21 years of age

**DOI:** 10.1530/ERC-26-0115

**Published:** 2026-09-21

**Authors:** Julio C Ricarte-Filho, Luz Castellanos, Steven G Waguespack, Julian Bryan, Mohammed Alshalalfa, Taylor B Cavazos, Yang Chen, Andrew Bauer, Joshua P Klopper, Richard T Kloos, Aime T Franco

**Affiliations:** ^1^ Division of Endocrinology and Diabetes, The Thyroid Center, Children’s Hospital of Philadelphia, Philadelphia, Pennsylvania, USA; ^2^ Department of Endocrine Neoplasia and Hormonal Disorders, The University of Texas MD Anderson Cancer Center, Houston, Texas, USA; ^3^ Molecular Diagnostics Laboratory, Division of Pathology and Laboratory Medicine, The University of Texas MD Anderson Cancer Center, Houston, Texas, USA; ^4^ Veracyte, Inc., South San Francisco, California, USA

**Keywords:** thyroid carcinoma, adolescent, *BRAF*, fusion, oncogenic driver

## Abstract

Although uncommon, thyroid nodules (TNs) in pediatric and young adult patients carry a higher malignancy risk and often present with a high burden of metastatic disease than in adults. The molecular features underlying this distinct clinical behavior remain unclear. We analyzed Afirma Genomic Sequencing Classifier (GSC) data from 283,621 TNs, comparing patients <21 and ≥21 years old. Cytology (Bethesda), GSC benign (B) vs suspicious (S) calls, and Afirma Xpression Atlas (XA) variant/fusion profiles were evaluated in GSC-S and Bethesda V/VI samples. Genome-wide expression was used to derive pathway signatures and thyroid cancer-related scores: *BRAF*-*RAS* score (BRS), ERK signaling score, follicular-to-mesenchymal transition (FMT) score, epithelial-to-mesenchymal transition (EMT) score, and thyroid differentiation score (TDS). Among 2,397 patients <21 years (median age: 18.9; 81.4% female) and 281,224 adults ≥21 years (median age: 59.8; 77.1% female), the <21-year samples showed more Bethesda V/VI cytology (14.5% vs 5.0%; *P* < 0.0001) and a lower GSC-B rate (43.5% vs 68.8%; *P* < 0.0001). In GSC-S samples, total variant detection was higher in the <21-year group (45.3% vs 37.4%), with enriched *BRAF* p.V600E, *TSHR*, and *DICER1* variants, while *HRAS* variants were more common in adults (all *P* < 0.01). Gene fusions involving *RET*, *NTRK3,* and *ALK* were enriched in the <21-year group (14.5% vs 5.5%; *P* < 0.0001). *TERT* promoter mutations were absent in <21-year GSC-S and Bethesda V/VI samples (vs 4.2% and 9.3% in adults). The GSC-S <21-year cohort showed cell-cycle pathway enrichment. The *RET/NTRK/ALK*-positive <21-year group demonstrated enrichment of angiogenesis and EMT pathways, higher ERK/EMT/FMT scores, and lower BRS/TDS vs genotyped-matched adults. These molecular differences provide mechanistic insight into the more invasive phenotype in pediatric and young adult TNs.

## Introduction

Thyroid nodules (TNs) are uncommon in patients <21 years but have a markedly higher malignancy risk (∼25%) than in adults (∼5–10%) (1). Patients <21 years also more often present with larger tumors, extrathyroidal extension, and both regional and distant metastatic disease; even in the presence of distant disease, the long-term prognosis is excellent (2, 3). Although disease-specific mortality is lower in patients <21 years, they face higher rates of treatment-related complications and recurrence (4, 5). Despite these well-recognized clinical differences, the molecular features that underlie the more invasive disease behavior in thyroid tumors from patients <21 years remain poorly characterized.

Papillary thyroid carcinoma (PTC) represents the most prevalent form of thyroid cancer (6). PTC is primarily driven by genetic alterations that lead to constitutive activation of the MAPK signaling pathway (7). While *BRAF* and *RAS* variants, as well as *RET*, *NTRK1/3*, and *ALK* fusions, occur in both patients <21 and >21 years, their prevalence differs. In adults, the most common alteration (58%) is the *BRAF* p.V600E variant (versus ∼25% in patients <21 years), whereas PTCs in patients <21 years are more frequently characterized by *RET*, *NTRK1/3*, and *ALK* fusions, which account for only ∼15% of adult cases (8, 9, 10). Beyond differences in prevalence, these genetic alterations also impact clinical behavior. PTCs in patients <21 years with kinase fusion oncogenes exhibit higher rates of distant metastases and persistent disease compared with adult PTCs with the same genotype (8, 9). In adults, *BRAF*-mutant PTCs are associated with a higher likelihood of metastatic progression and radioiodine (RAI)-refractory behavior (11, 12, 13). By contrast, in patients <21 years, this variant is predominantly associated with regional metastasis and is generally linked to a lower risk of distant metastasis (3, 9, 14, 15). However, the extent to which *BRAF* p.V600E is an independent predictor of prognosis in PTC remains debated (16). Clarifying the pathways and processes that drive age-dependent phenotypic differences, particularly within the same genotype, is critical for advancing disease management.

The pre-operative use of next-generation sequencing panels for indeterminate TNs (ITNs) improves diagnostic accuracy and informs surgical management decisions (17, 18). The Afirma Genomic Sequencing Classifier (GSC) is an exome-enriched RNA sequencing assay that sequences over 21,000 gene transcripts, allowing for the detection of gene fusions, sequence variants, gene expression, and pathway enrichment analysis (19). Its primary objective is to classify indeterminate thyroid nodules as benign or suspicious, ruling out a cancer diagnosis in approximately two-thirds of ITNs and reducing unnecessary surgeries (17, 20). In addition, the extensive molecular data from Afirma’s vast biorepository enables the investigation of molecular changes linked to specific genotypes, ultimately improving our understanding of disease biology (21, 22).

To date, comprehensive comparative genomic analyses of thyroid nodules across age groups remain limited. In this study, we report Afirma GSC data from 283,621 thyroid nodules, aiming to assess molecular differences in nodules from pediatric and young adult (PYA) patients (<21 years) compared with those from adults (≥21 years). Our findings from cytological and mutational analyses underscore the clinical and molecular distinctions between PYA thyroid nodules and those occurring in adults. Furthermore, genome-wide differential gene expression profiling identifies pathway- and network-level differences associated with these groups, highlighting key molecular distinctions between thyroid carcinomas in PYA and adult patients.

## Materials and methods

### Thyroid nodule evaluation

**Figure 1:**
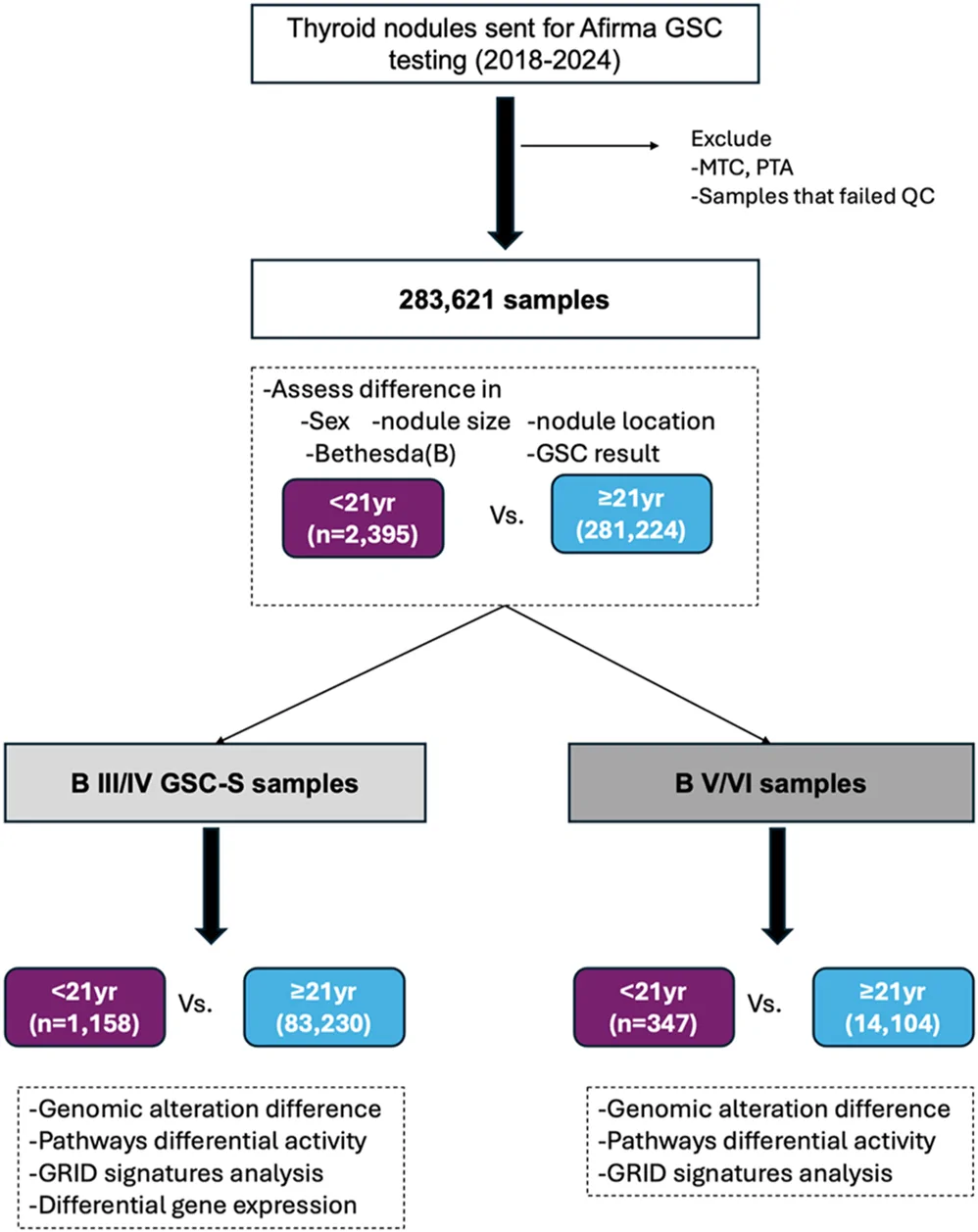
Flow chart of the cohort used, and analyses conducted. GSC, genomic sequencing classifier, MTC, medullary thyroid carcinoma, PTA, parathyroid adenoma. A full color version of this figure is available at https://doi.org/10.1530/ERC-26-0115.

Fine-needle aspiration (FNA) specimens are classified according to the Bethesda System for Reporting Thyroid Cytopathology (TBSRTC) into 1 of 6 diagnostic categories with a range of malignancy risk: I, nondiagnostic (mean: 14%, range: 0–33); II, benign (6%; 0–27); III, atypia of undetermined significance (28%; 11–54); IV, follicular neoplasm (50%; 28–100); V, suspicious for malignancy (81%; 40–100); and VI, malignancy (98%; 86–100) (23). Analysis of 283,621 consecutive thyroid nodules with clinical Afirma GSC results from 2018 to 2024 with Bethesda III–VI cytology by age (<21 and ≥21 years), cytology category (BIII–VI), sex, GSC-suspicious (S) rate, genomic alterations, and molecular pathways differential activity was performed in this retrospective observational cohort study (Fig. 1). Consecutive Afirma samples were sent at the discretion of the ordering clinician, almost exclusively from a variety of practices including endocrine clinics, surgery clinics, and radiology centers from within the United States. Cytology was read either at a local pathology practice or, when requested, at Thyroid Cytopathology Partners (USA).

### Variant, fusion, and molecular pathways differential analysis

The relative frequencies of genomic alterations (variants and fusions) from the Afirma Xpression Atlas (24) (XA) portion of the Afirma GSC were evaluated by patient age from ITN GSC-S samples from those <21 and ≥21 years and BV/VI TNs. Our analysis was limited to the most frequently altered genes and most common fusions. For characterizing molecular differences in pathways, 50 gene expression signatures from the molecular signatures database (MSigDB) and 5 thyroid cancer-related signature scores (21) were evaluated. These include activity scores of 50 hallmark cancer pathways (25), *BRAF*-like to *RAS*-like score (BRS), ERK signaling score, thyroid differentiation score (TDS), epithelial-to-mesenchymal transition (EMT) score, and follicular-to-mesenchymal transition (FMT) score. Logistic regression and Wilcoxon rank-sum test were used to assess the significance of the molecular differences between age groups (<21 vs ≥21 years).

### Differential gene expression and gene set enrichment analysis

To identify molecular differences at the gene level, transcriptome-wide differential expression analysis using 26,268 genes was conducted to identify genes upregulated and downregulated in patients <21 and ≥21 years using Wilcoxon rank-sum test. Top genes were used for further downstream functional analysis. g:Profiler online tool (https://biit.cs.ut.ee/gprofiler/gost) was used to identify biological pathways/gene ontology biological processes (GO/BP) enriched in upregulated and downregulated genes in <21-year nodules (26).

### Statistical analysis

Wilcoxon rank-sum test was used to determine the association between continuous variables and age groups (<21 vs ≥21 years). Chi-square tests were used to associate categorical variable and age groups. Logistic regression was used to calculate the odds ratio (OR) associating the <21-year age group (dependent variable) with high pathway scores, where high was defined as the top 10% or 25% of scores (independent variables). Logistic regression modeling was conducted within several molecularly homogeneous groups to adjust for potential confounding by molecular variables. The models were not adjusted for additional potential confounders, including age, sex, cytology category, or nodule size; therefore, residual confounding by these and other unmeasured variables cannot be excluded. Multiple testing *P* value correction was performed using the Benjamini–Hochberg (BH) procedure. All statistical analyses were conducted with R version 4.2.0 (R Foundation for Statistical Computing, Austria).

### Institutional review board approval

The evaluated data were free of protected health information, and the research protocol was approved by the WCG IRB DHF 005-045P.

## Results

There were 2,397 patients <21 years (median age: 18.9 years (IQR: 17.1–21; 81.4% female)) and 281,224 patients ≥ 21 years (median age: 59.8 years (IQR: 47.0–69.8; 77.1% female)) (Table 1). There were significantly more BV/VI samples in the <21-year vs ≥21-year group (14.5% vs 5%, *P* < 0.0001) (Table 1). The GSC-B rate was significantly lower in the <21-year (43.5%) than in the ≥21-year group (68.8%, *P* < 0.0001) (Table 1). There was no difference in nodule size, nodule location, Bethesda, and GSC-B rate by age and sex within the <21-year group. However, within the ≥21-year group, nodules from male were more associated with BV/VI and higher rate of GSC-S (Supplementary Table S1 (see section on Supplementary materials given at the end of the articles)). Patients <15 years showed a similar clinical profile to the overall <21-year group, and there was no difference by sex within <15 years group (Supplementary Table S2 and 3).

**Table 1:** Clinical characteristics of TNs in the <21-year and ≥21-year cohorts.

	<21 years	≥21 years	*P* value
Total	2,397	281,224	
Age in years			
Median (IQR)	18.9 (17.1–21)	59.8 (47–69.8)	
<15 years	253 (10.5%)		
(15–21) years	2,144 (89.5%)		
Sex			
Male	446 (18.6%)	64,370 (22.9%)	< 0.0001*
Female	1,951 (81.4%)	216,799 (77.1%)	
Nodule size in cm median (IQR)	2.3 (1.5–3.3)	2.2 (1.6–3.1)	n.s.
Nodule location			
Isthmus	108 (4.5%)	13,164 (4.7%)	n.s.
Right lobe	1,232 (51.4%)	137,904 (49%)	
Left lobe	981 (40.9%)	121,743 (43.3%)	
Bethesda			
ITN III	1,603 (66.9%)	222,846 (79.2%)	< 0.0001*
ITN IV	447 (18.6%)	44,274 (15.7%)	
V and VI	347 (14.5%)	14,104 (5%)	
GSC within ITNs			
GSC-S	1,158 (56.5%)	83,230 (31.1%)	< 0.0001*
GSC-B	892 (43.5%)	183,809 (68.8%)	

* Chi-square test; n.s. not significant (adjusted *P* > 0.05).

We next characterized the Afirma XA results in ITN GSC-S and BV/VI separately to reduce the impact of BV/VI on the variant and fusion frequency rates. Overall, there were more total variants detected on Afirma XA in the <21-year vs ≥21-year group (45.3% vs 37.4%, *P* < 0.0001) in ITN GSC-S samples (Table 2). Variants that were more prevalent in the <21-year than the ≥21-year group included *BRAF* p.V600E (hereafter *BRAF*; 9.7% vs 7.6%), *TSHR* (2.4% vs 1.2%), and *DICER1* (9.7% vs 1.5%) (*P* < 0.01 for all). *HRAS* variants were enriched in the ≥21-year group (8.6% vs 5.6%, *P* = 0.001). A similar pattern was observed for *SPOP* (0.9% vs 0%, *P* = 0.006). Within a subset of ITN GSC-S that had the *TERT* promoter (*TERTp*) mutation profiles (*n* = 122 for <21 and *n* = 10,918 for ≥21), there was no *TERTp* mutation detected in the <21-year group compared with 4.2% in the ≥21-year group (*P* = 0.038). There was a greater proportion of fusions in the <21-year group (14.5%) than in the ≥21-year group (5.5%) (*P* < 0.0001) with the most significant differences in *NTRK3 (*6% vs 1%, *P* < 0.0001, *ETV6::NTRK3* most commonly), followed by *RET (CCDC6::RET* most commonly*)* and *ALK (STRN::ALK* most commonly*)* fusions (Table 2). Other rarer fusions identified are listed in Table 2.

**Table 2:** Most frequent XA genomic alterations (variants and fusions) across age groups in ITN GSC-S.

		<21 years (*n* = 1,158)	≥21 years (*n* = 83,230)	*X*^2^ *P* value*
	**Any variant alterations**	**524 (45.3%)**	**31,097 (37.4%)**	**<0.0001**
Variants	*BRAF* p.V600E	112 (9.7%)	6,288 (7.6%)	0.018
	*BRAF* p.K601E	15 (1.3%)	913 (1.1%)	n.s.
	*NRAS*	176 (15.2%)	11,472 (13.8%)	n.s.
	*DICER1*	112 (9.7%)	1,270 (1.5%)	<0.0001
	*HRAS*	65 (5.6%)	7,195 (8.6%)	0.001
	*TSHR*	28 (2.4%)	1,018 (1.2%)	0.001
	*KRAS*	17 (1.5%)	1,421 (1.7%)	n.s.
	*OBSCN*	4 (0.3%)	134 (0.2%)	n.s.
	*PIK3CA*	3 (0.3%)	108 (0.1%)	n.s.
	*SPOP*	0	727 (0.9%)	0.006
	*EIF1AX*	0	463 (0.6%)	0.04
	*JAK2*	0	204 (0.2%)	n.s.
	*TP53*	0	148 (0.2%)	n.s.
	*FAT1*	0	104 (0.1%)	n.s.
	*EZH1*	0	37 (<0.1%)	n.s.
	*PTEN*	0	21 (<0.1%)	n.s.
Fusions	**Any fusion**	**168 (14.5%)**	**4,593 (5.5%)**	<0.0001
	*Any RET/ALK/NTRK fusion*	115 (9.9%)	1,671 (2%)	<0.0001
	*NTRK3*	69 (6%)	814 (1%)	<0.0001
	*PAX8*	40 (3.5%)	2,071 (2.5%)	n.s.
	*RET*	30 (2.6%)	565 (0.7%)	<0.0001
	*ALK*	13 (1.1%)	188 (0.2%)	<0.0001
	*BRAF*	7 (0.6%)	418 (0.5%)	n.s.
	*NTRK1*	3 (0.3%)	105 (0.1%)	n.s.
TERT	**Total samples with *TERT* mutational profile**	**122**	**10,918**	
	*TERT (C228T, C250T)*	0	455 (4.2%)	0.038

* *P* values adjusted for multiple testing. Bold entries indicate aggregate analyses comparing the prevalence of any variant alteration, any fusion event, or TERT promoter mutations between age groups.

n.s. not significant (adjusted *P* > 0.05).

In BV/VI samples, there were more XA variants detected in the ≥21-year group compared with the <21-year group (66.9% vs 58.5%, *P* = 0.006), but the fusion rate was higher in the <21-year group (20.7% vs 7.9%, *P* < 0.0001, Table 3). *CCDC6::RET* and *ETV6::NTRK3* were, again, the most overrepresented fusions. Again, there were no *TERTp* mutations detected in <21 years compared with 9.3% in ≥21 years in BV/VI samples with *TERTp* mutation profiles (*n* = 80 for <21 and *n* = 3,381 for ≥21) (Table 3). Minimal genomic differences were observed comparing <15 vs 15- to 21-year groups in both GSC-S and BV/VI samples (Supplementary Table S4 and 5).

**Table 3:** Most frequent XA genomic alterations (variants and fusions) across age groups in BV/VI TNs.

		<21 years (*n* = 347)	≥21 years (*n* = 14,104)	*X*^2^ *P* value*
	**Any variant alterations**	**203 (58.5%)**	**9,440 (66.9%)**	**0.006**
Variants	*BRAF* p.V600E	182 (52.4%)	8,546 (60.6%)	0.01
	*BRAF* p.K601E	0	18 (0.1%)	n.s.
	*NRAS*	9 (2.6%)	286 (2%)	n.s.
	*DICER1*	4 (1.2%)	99 (0.7%)	n.s.
	*HRAS*	2 (0.6%)	144 (1%)	n.s.
	*TSHR*	4 (1.2%)	122 (0.9%)	n.s.
	*KRAS*	1 (0.3%)	82 (0.6%)	n.s.
	*OBSCN*	0	36 (0.3%)	n.s.
	*PIK3CA*	2 (0.6%)	72 (0.5%)	n.s.
	*SPOP*	0	68 (0.5%)	n.s.
	*EIF1AX*	0	26 (0.2%)	n.s.
	*JAK2*	0	15 (0.1%)	n.s.
	*TP53*	2 (0.6%)	36 (0.3%)	n.s.
	*FAT1*	0	24 (0.2%)	n.s.
	*EZH1*	0	10 (0.1%)	n.s.
	*PTEN*	1 (0.3%)	2 (<0.1%)	n.s.
Fusions	**Any fusion**	**72 (20.7%)**	**1,120 (7.9%)**	<0.0001
	*Any RET/ALK/NTRK fusion*	67 (19.3%)	771 (5.5%)	<0.0001
	*NTRK3*	21 (6.1%)	200 (1.4%)	<0.0001
	*PAX8*	1 (0.3%)	112 (0.8%)	n.s.
	*RET*	43 (12.4%)	482 (3.4%)	<0.0001
	*ALK*	1 (0.3%)	41 (0.3%)	n.s.
	*BRAF*	2 (0.6%)	160 (1.1%)	n.s.
	*NTRK1*	2 (0.6%)	49 (0.3%)	n.s.
TERT	**Total samples with *TERT* mutational profile**	**80**	**3,381**	
	*TERT (C228T, C250T)*	0	313 (9.3%)	0.008

* *P*-values adjusted for multiple testing. Bold entries indicate aggregate analyses comparing the prevalence of any variant alteration, any fusion event, or TERT promoter mutations between age groups.

n.s. not significant (adjusted *P* > 0.05).

**Figure 2:**
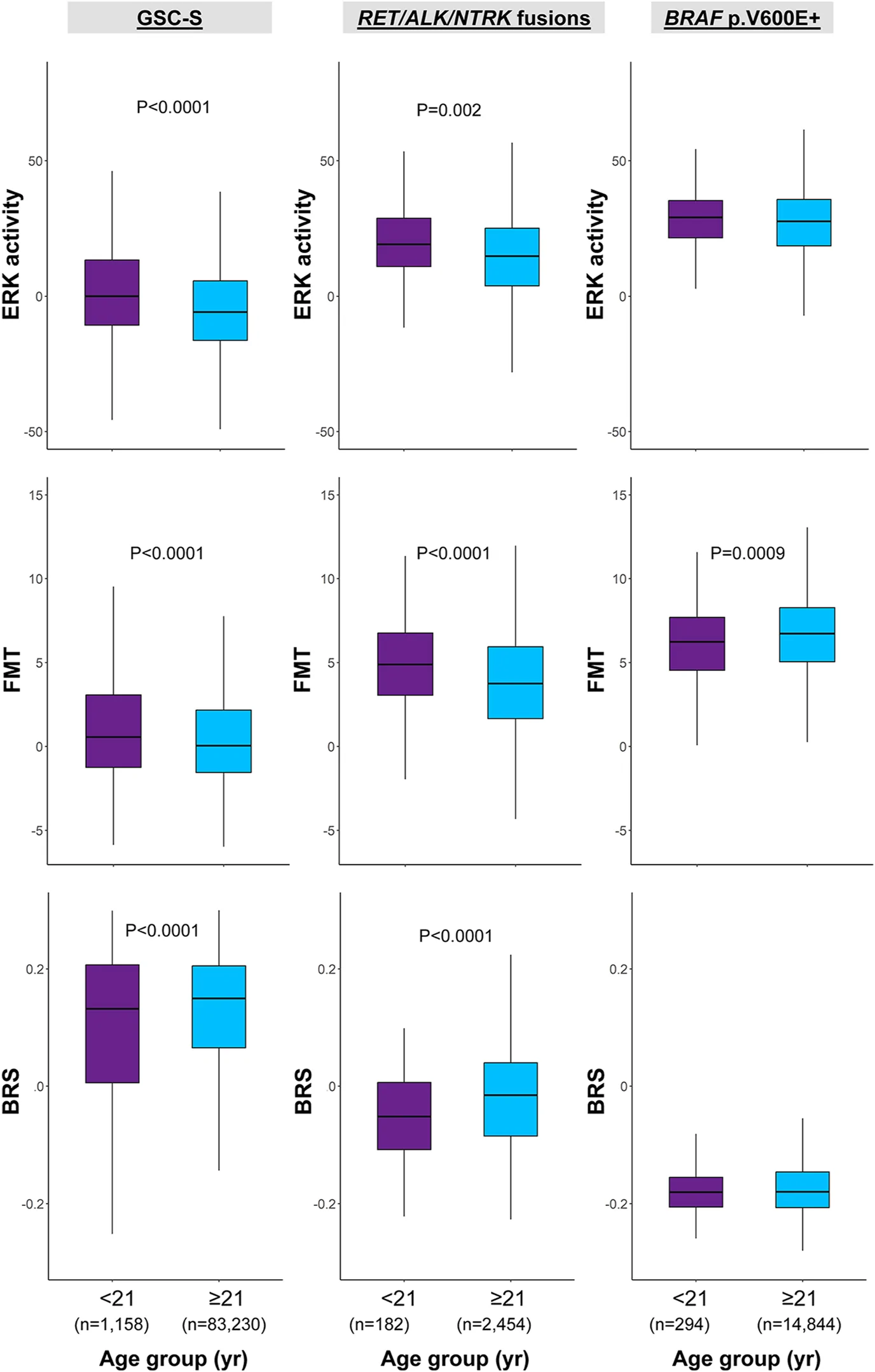
Relative activity of multiple thyroid signatures of follicular–mesenchymal transition (FMT), BRAF-RAS score (BRS), and ERK activity in the younger (<21) and older (≥21) patients across different subset of patients. A full color version of this figure is available at https://doi.org/10.1530/ERC-26-0115.

Expression signatures for FMT, EMT, TDS, BRS, and ERK activity scores were characterized between <21 and ≥21 years across different sample subsets (GSC-S, BV/VI, *BRAF*+, *RET/NTRK/ALK* fusion-positive) (Figs 2 and S1–2). These scores were statistically different between age groups in GSC-S and *RET/NTRK/ALK* fusion-positive samples. Patients <21 years have higher ERK activity, FMT, and EMT, and lower BRS (*BRAF*-like). Within *BRAF*-mutant samples, there were no differences except for FMT that was lower in the <21-year group (*P* = 0.0009). TDS did not show a consistent trend: it was higher in <the 21-year group in GSC-S (*P* < 0.0001) and lower in the <21-year group in samples with fusions (*P* = 0.002, Supplementary Fig. S1) but without a difference in samples with *BRAF* mutation. Within BV/VI samples, only ERK score was higher in the <21-year cohort, and the other scores were not different (*P* = 0.007, Supplementary Fig. S2A).

**Figure 3:**
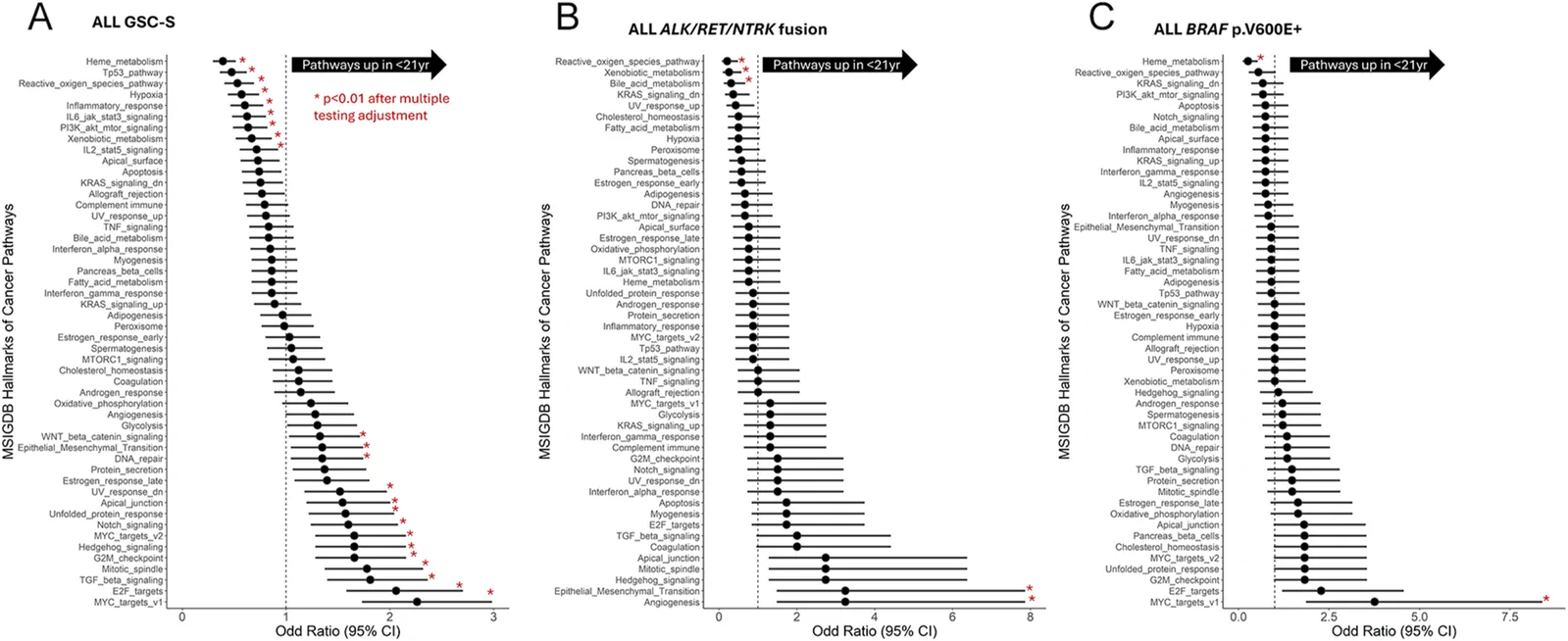
Forest plot of hallmarks of cancer signatures associating signature activity with age group across different subsets of patients: (A) all GSC-S, (B) RET/NTRK/ALK fusions, and (C) *BRAF* p.V600E. Odds ratio (OR) was calculated by splitting the pathway activity into high (top25%) vs low. The dashed vertical line represents where signatures are underexpressed (to the left of the line) or overexpressed (to the right of the line) in the <21-year group. The asterisk represents signatures that are significantly under- or overexpressed after multiple testing *P* value correction. *P*-values were generated from the Wilcoxon rank-sum test. A full color version of this figure is available at https://doi.org/10.1530/ERC-26-0115.

When characterizing hallmarks of cancer signatures across age groups using logistic regression with using the top 25% as high score, the <21-year group had increased activity of cell-cycle-related pathways (E2F pathway, mitotic spindle, G2M checkpoint) and decreased activity of inflammation and immune modulation pathways in GSC-S samples (*P* < 0.01, Fig. 3A). Within samples with *RET/NTRK/ALK* fusions (including both GSC-S and BV/VI), the <21-year group had higher EMT and angiogenesis (*P* < 0.01, Fig. 3B). A similar trend was observed when defining high pathway activity as the top 10% (Supplementary Fig. S3). In addition, within samples with *BRAF* mutation (including both GSC-S and BV/VI), the <21-year group had higher MYC scores (*P* < 0.01, Fig. 3C). Within BV/VI samples, there was no difference in the pathway activity between the age groups (Supplementary Fig. S2B).

**Figure 4:**
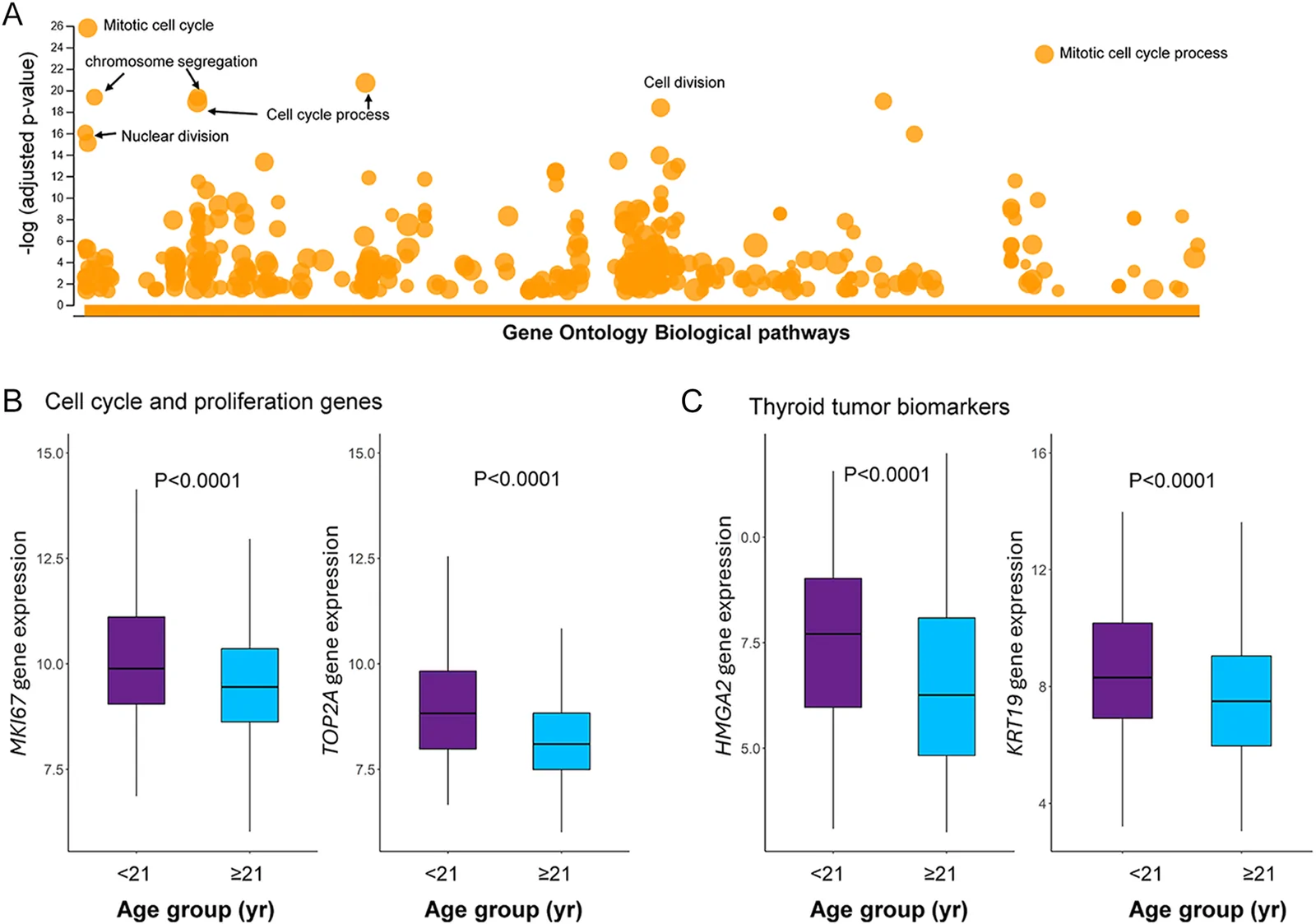
Gene set enrichment analysis of GO:BP terms in genes upregulated in the <21-year cohort. (A) The top 500 upregulated genes were highly enriched in cell cycle genes. (B) Key cell cycle and cell proliferation markers (*TOP2A*, *MKI67*) were among the top upregulated genes. (C) Key thyroid cancer diagnostic biomarkers are highly upregulated in the <21-year cohort. A full color version of this figure is available at https://doi.org/10.1530/ERC-26-0115.

Finally, we conducted differential gene expression analysis between the <21-year and ≥21-year groups. A total of 4,585 genes were differentially expressed (BH-corrected *P* < 0.001), of which 2,355 were upregulated in <21 years. We conducted pathway enrichment analysis (using gene ontology biological processes terms) on the top 500 upregulated genes using the g:Profiler tool. Enrichment analysis showed that the <21-year samples were highly enriched with cell-cycle-related pathways (Supplementary Table S6), indicating a higher proliferation rate (Fig. 4A). The cell proliferation markers (*TOP2A* and *MKI67*) were among the most upregulated genes in the <21-year group (*P* < 0.0001, Fig. 4B). Key thyroid carcinoma diagnostic biomarkers, such as *KRT19* and *HMGA2*, were among the top upregulated genes in the <21-year cohort (*P* < 0.0001, Fig. 4C).

## Discussion

In this study, we have investigated the cytological and molecular differences between thyroid nodules sent for molecular testing as part of routine care for patients <21 and ≥21 years. We show that clinicians opt for testing in a relatively higher proportion of BV/VI nodules in the <21-year-old patients regardless of sex. For those patients <21 years, the GSC-S call rate is significantly higher than those the ≥21-year group, which is consistent with the well-described higher rates of malignancy in pediatric and young adult ITNs (27, 28). GSC-S samples from the <21-year group showed a higher frequency of both pathogenic variants and fusions. Combined, *RET, NTRK1/3,* and *ALK* fusions and *BRAF* mutation were twice as common in the <21-year group compared with the ≥21-year and may contribute to the elevated malignancy risk observed in ITNs within this population. We observed increased *BRAF* mutation prevalence in GSC-S samples and reduced prevalence in BV/VI samples from patients <21 years compared with those ≥21 years. This is consistent with prior studies and corroborated by public data from AACR GENIE, where primary thyroid carcinomas the ≥21-year group display a higher frequency of *BRAF* mutations (29). The observations of a higher rate of *BRAF* p.V600E and *RET*, *NTRK1/3*, and *ALK* fusions support the clinical utility of performing somatic oncogene testing in patients <21 years with indeterminate cytology.

Nodules from the <21-year cohort demonstrated higher ERK and FMT scores, along with lower BRS and elevated TDS, indicating enhanced MAPK signaling activity and greater metastatic potential, while maintaining a relatively differentiated state compared with nodules from the ≥21-year cohort. The enhanced thyrocyte differentiation in the <21-year cohort ITNs is consistent with the higher RAI responsiveness in young PTC patients, offering a potential explanation for the characteristically favorable prognosis of PYA thyroid carcinomas (30, 31).

The significance of these transcriptional scores becomes clearer when stratified by genotype, revealing genotype-specific programs and pathway activation patterns that may contribute to distinct biological and clinical phenotypes. When focusing solely on cases harboring *RET/NTRK/ALK* fusions, the <21-year cohort showed exclusive enrichment of two hallmark cancer pathways, EMT and angiogenesis, compared with *RET/NTRK/ALK*+ nodules from the ≥21-year cohort. Both EMT and tumor-induced lymphangiogenesis and angiogenesis are critical steps in metastatic progression (32, 33) and may help explain why nodal and pulmonary metastases occur more frequently in *RET/NTRK/ALK*-driven PYA tumors than in genotype-matched adult tumors (9). Furthermore, transcriptional profiling indicates increased MAPK signaling (↑ERK) and reduced thyroid differentiation (↓TDS) in *RET/NTRK/ALK*-driven nodules from the <21-year cohort relative to the ≥21-year cohort, suggesting a potential molecular basis for their association with more invasive disease and a high rate of persistent disease in the <21-year cohort with pulmonary metastasis even in the absence of additional genetic alterations (3, 9, 34, 35, 36, 37). Notably, differences in MAPK signaling do not fully account for the differences in metastatic behavior observed in PYA cases. Despite exhibiting lower MAPK output, *RET/NTRK/ALK*-positive PYA nodules are known to exhibit enhanced metastatic potential compared with *BRAF*-mutant (9). This likely reflects the broader signaling repertoire of receptor tyrosine kinase fusions, which can concurrently activate MAPK, PI3K/AKT, and possibly additional pathways driving EMT and pro-angiogenic regulatory networks.

Unlike the *RET/NTRK/ALK* subset, *BRAF*-driven nodules showed no age-related differences in ERK, BRS, TDS, or EMT scores, with the sole exception of elevated FMT scores in adult nodules. Interpretation of this observation is challenging due to the clinical heterogeneity of adult *BRAF*-driven PTC, which can display variable behaviors and differentiation states (38), likely driven by the acquisition of additional genetic events during progression (39, 40, 41). No PYA samples in our cohort harbored *TERT* promoter mutations. Although screening was performed in only a limited subset of the entire cohort and, thus, requires cautious interpretation, this finding is consistent with prior reports indicating that such mutations are exceedingly rare in PYA cases (42). The occurrence of these mutations exclusively in the adult cases adds a potential confounder since the coexistence of *TERT* promoter and *BRAF* mutation is linked to poorer outcomes in PTC, characterized by higher MAPK output and lower differentiation scores (8, 43). Future studies that distinguish *BRAF/TERT* co-mutant cases from those harboring *BRAF* mutations alone will be essential to enhance the resolution of age-related differences in *BRAF*-mutant nodules.

Genome-wide expression and hallmark enrichment analyses revealed that cases <21 years show significant enrichment of MYC, E2F, and cell cycle pathways, along with upregulation of proliferation-related genes such as *MKI67* and *TOP2A,* indicating enhanced proliferative signaling via the MYC>E2F>cell cycle axis. In the <21-year cohort, *HMGA2* and *KRT19*, two genes with diagnostic relevance in thyroid carcinoma, were prominently upregulated (44, 45). In addition, both genes have also been shown to enhance thyroid carcinoma invasiveness and metastatic potential (46, 47). The upregulation of these genes in the <21-year cohort may reflect the higher prevalence of *BRAF* mutations and *RET/NTRK/ALK* fusions, alterations that are associated with increased expression of these genes compared with RAS-like mutations (8).

The molecular landscape of thyroid nodules in individuals <21 years has been explored by Gallant *et al.* using the ThyroSeq v3 DNA/RNA next-generation sequencing assay (48). This was a retrospective study primarily analyzing molecular results of 95 formalin-fixed, paraffin-embedded histopathology samples from 79% females with a median age of 16.3 years. Since the final pathology was known, mutations could be correlated with final histology. In 50 malignant samples, there was a predominance of fusions, primarily *CCDC6::RET* (48%), *NCOA4::RET* (28%), and *ETV6::NTRK3* (7%). Only 44 samples had pre-operative Bethesda criteria described, and 17 (39%) were BVI and 12 (27%) were BII. By contrast, this study’s samples were all from pre-operative FNA samples with a much larger sample size, and the majority were from ITNs, as might be expected from the routine clinical use of molecular testing. In addition, the Afirma exome-enriched RNAseq assay allows for the interrogation of hallmarks of cancer signatures and DGE analyses.

This study is subject to potential selection bias, as samples were submitted for Afirma testing at the discretion of the ordering clinician, likely enriching for cytologically indeterminate nodules. In addition, the analysis was limited to nodules with surgically actionable Afirma or cytology results (GSC-S or Bethesda V/VI), which may influence the observed differences between groups. Accordingly, our findings should be interpreted within the context of this selected cohort and may not be generalizable to all thyroid nodules. The <21-year cohort is relatively old, with a median age of almost 19 years, and primarily reflects adolescents and young adults, limiting our ability to stratify molecular findings in younger pediatric patients (e.g. pre-pubertal). Although stratified analyses were performed, residual confounding by factors such as sex, cytology category, nodule size, and other unmeasured variables cannot be excluded as statistical analyses were otherwise unadjusted. In addition, final histopathology is unknown; thus, we cannot correlate the molecular findings with tumor stage or longer-term follow-up (i.e. recurrence or mortality). Important future studies will include correlations with final histopathology and determinants of why, despite molecular findings of increased invasiveness in samples from the <21-year cohort, clinical outcomes for PYA thyroid carcinoma patients are generally excellent. In addition, data analysis for the GSC-B samples will be essential to determine the potential clinical utility of using the gene sequencing classifier to avoid surgery in patients <21 years with indeterminate cytology.

In summary, this study highlights the cytological and molecular distinctions that shape the unique clinical profiles of thyroid tumors in PYA compared with adults. Our findings support the higher malignancy rate in PYA nodules, reflected by a higher rate of GSC-S in nodules with indeterminate cytology from the <21-year cohort. At the molecular level, PYA nodules exhibited enrichment of proliferative pathways and a higher prevalence of kinase fusions, particularly *RET* and *NTRK3*, as well as *DICER1* mutations. By delineating pathways enriched in fusion-driven PYA tumors, including angiogenesis, EMT, ERK signaling, and reduced thyroid differentiation scores, this work provides insights into molecular features associated with age-related clinical differences in patients with fusion-driven PTC.

## Supplementary materials





## Declaration of interest

MA, JPK, TBC, and YC are employees and equity owners of Veracyte, Inc. RTK was a previous employee and equity owner of Veracyte, Inc. All other authors declare that they have no conflicts of interest to disclose.

## Funding

This work did not receive any specific grant from any funding agency in the public, commercial, or not-for-profit sector.

## Data availability

Original data generated and analyzed during this study are included in this published article.

## Ethics statement

The evaluated data were free of protected health information, and the research protocol was approved by the WCG IRB DHF 005-045P.
