# Supplementary material for: Distinct molecular profiles of indeterminate and malignant thyroid nodules in patients under 21 years of age
Source: Endocr Relat Cancer. 2026 Sep 21;33(9):e260115. doi: 10.1530/ERC-26-0115 (PMC13615848; doi:10.1530/ERC-26-0115)
Supplement: Supplementary file 1 [file ERC-26-0115_supplementary_figures.pdf]

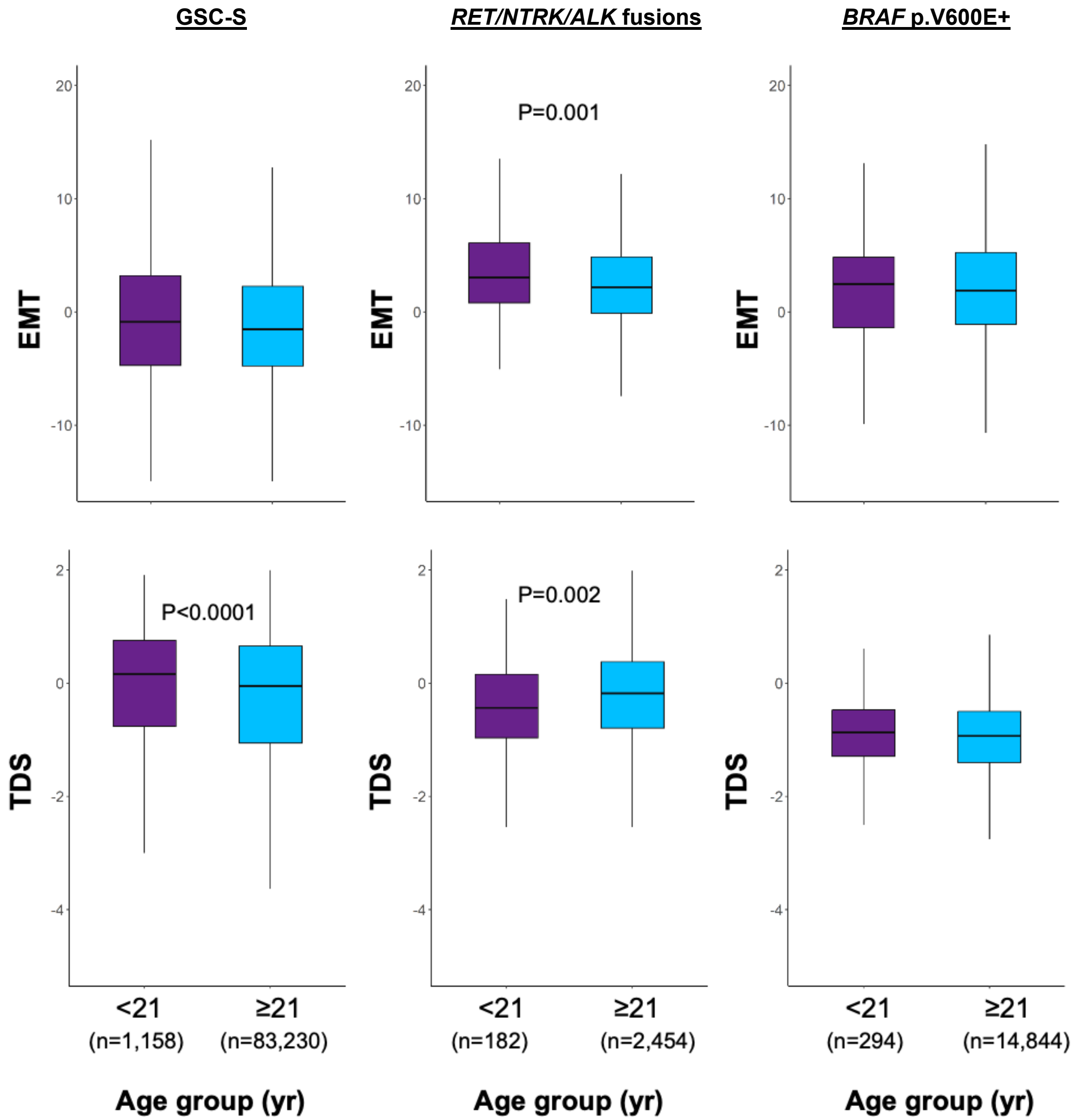

Figure S1: Relative activity of multiple thyroid signatures of Epithelial-Mesenchymal Transition (EMT) and Thyroid Differentiation Score (TDS) in younger (< 21yr) and older (≥ 21) patients across different subset of patients.

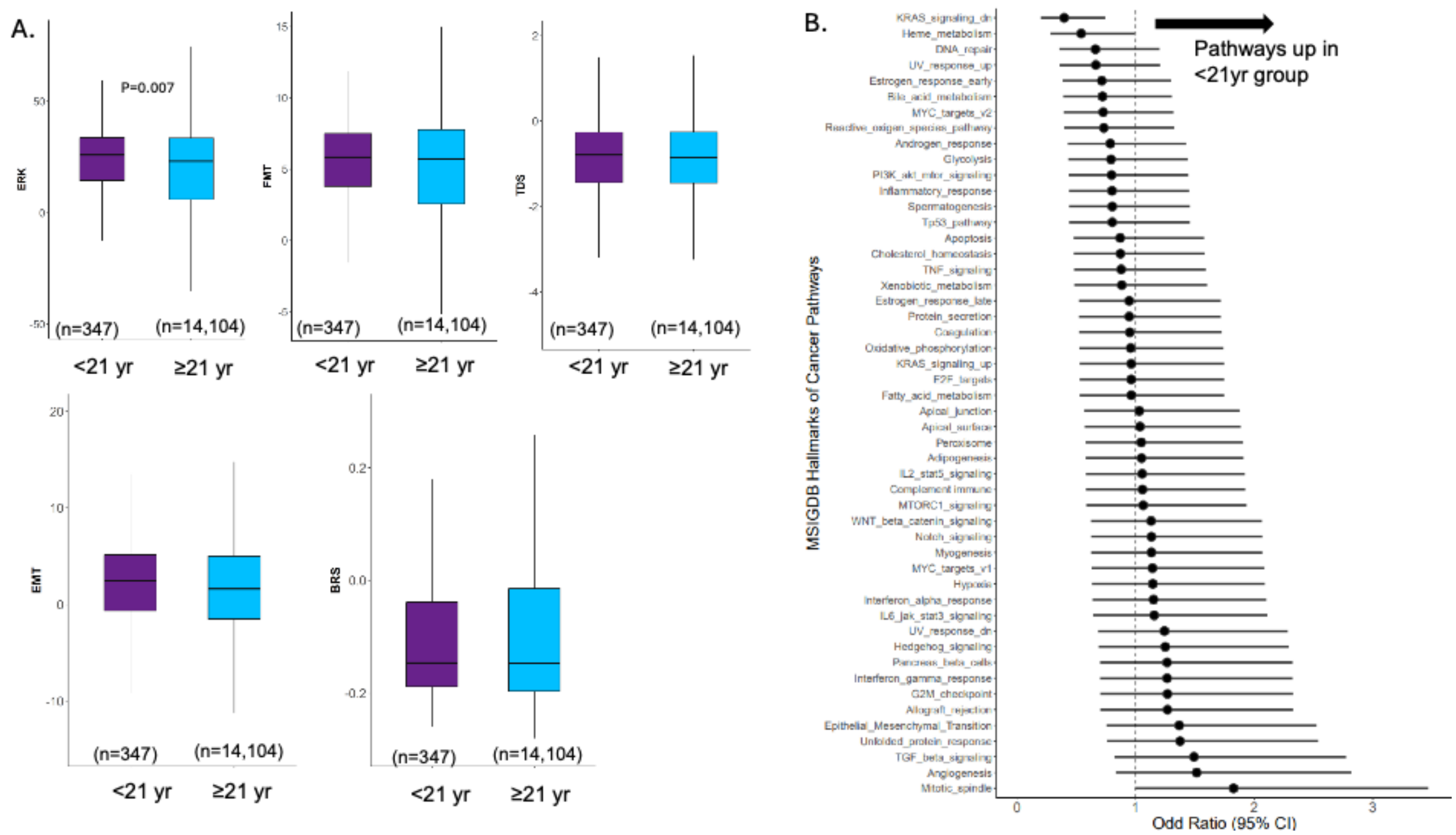

<u>Figure S2</u>: Molecular differences between <21 and ≥ 21 in B V/VI samples. (A) Activity of thyroid signatures and (B) forest plot of hallmarks of cancer and in < 21 vs ≥ 21 yrs in samples with BV/VI. The observed difference is less evident in this group compared to the clear difference in GSC-S and *RET/NTRK/ALK* fusions samples.

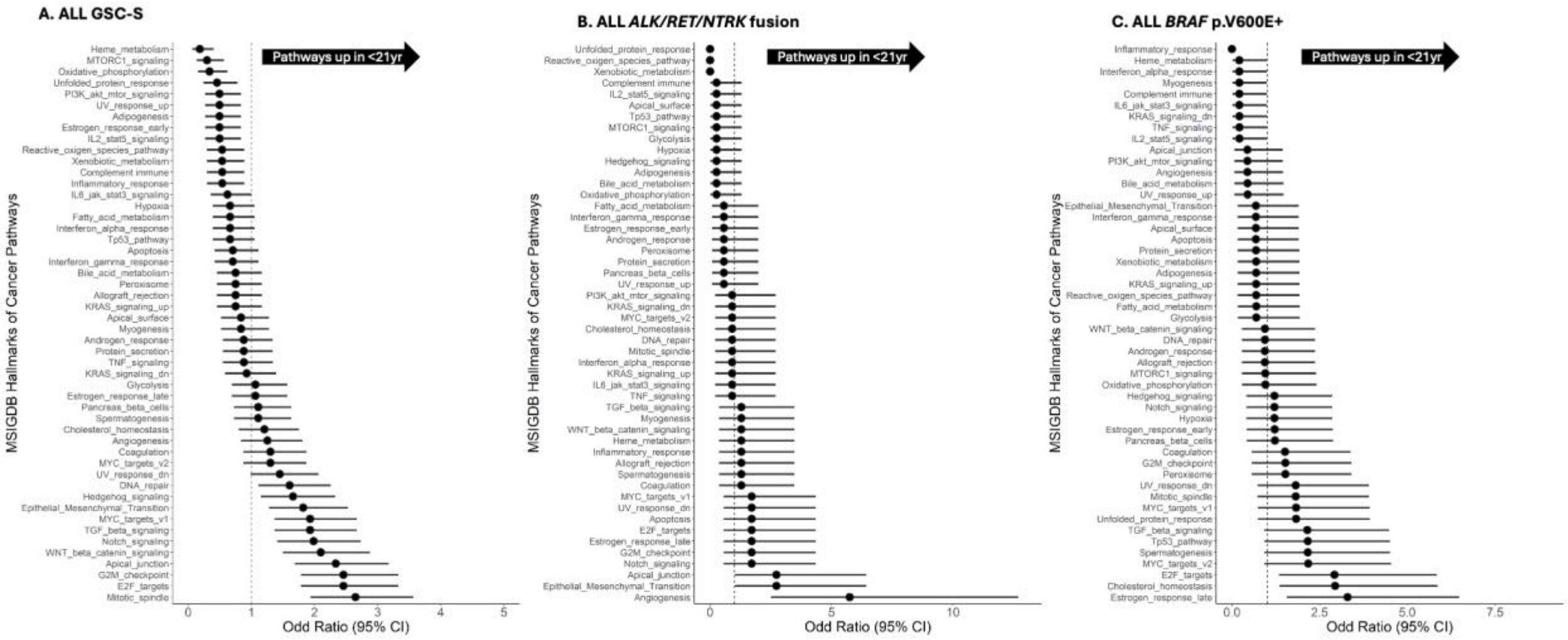

Figure S3: Forest plot of hallmarks of cancer signatures associating signature activity with age group across different subsets of patients (A. All GSC-S, B. RET/NTRK/ALK fusions, C. *BRAF* p.V600E). Odd Ratio (OR) was calculated by splitting the pathway activity into high (top10%) vs others.
